# The typically developing paediatric foot: how flat should it be? A systematic review

**DOI:** 10.1186/s13047-017-0218-1

**Published:** 2017-08-15

**Authors:** Hayley Uden, Rolf Scharfbillig, Ryan Causby

**Affiliations:** School of Health Sciences, University of South Australia, Adelaide, South Australia, 5001 Australia

**Keywords:** Children, Flat foot, Foot development, Foot posture, Paediatric foot, Systematic review

## Abstract

**Background:**

All typically developing children are born with flexible flat feet, progressively developing a medial longitudinal arch during the first decade of their lives. Whilst the child’s foot is expected to be flat, there is currently no consensus as to *how flat* this foot should be. Furthermore, whilst feet are observed to decrease in flatness with increasing age, it is not known *how flat* they should be at each age increment. The objective of this systematic review is to define the postural characteristics of the ‘typically’ developing paediatric foot.

**Methods:**

The PRISMA protocol was applied to compare all data currently published describing the typical development of the paediatric foot. The Epidemiological Appraisal Instrument (EAI) was used to assess the risk of bias of the included studies.

**Results:**

Thirty four epidemiological papers pertaining to the development of the paediatric foot were graphically compared. Sixteen different foot posture assessments were identified of which footprint based measures were the most reported outcome.

**Conclusion:**

Firstly, the use of the term *normal* in relation to foot posture is misleading in the categorisation of the paediatric foot, as indeed a flat foot posture is a normal finding at specific ages. Secondly, the foot posture of the developing child is indeed age dependent and has been shown to change over time. Thirdly, no firm conclusion could be reached as to which age the foot posture of children ceases to develop further, as no two foot measures are comparable, therefore future research needs to consider the development of consensus recommendations as to the measurement of the paediatric foot, using valid and reliable assessment tools.

**Electronic supplementary material:**

The online version of this article (doi:10.1186/s13047-017-0218-1) contains supplementary material, which is available to authorized users.

## Background

All typically developing children are born with flexible flat feet, progressively developing a medial longitudinal arch during the first decade of life [1, 2]. This trend of reducing flat foot with increasing age is consistently noted within the literature [1, 3, 4]. Despite flat feet being a typical developmental occurrence, it is still a frequent reason for which parents seek paediatric medical opinion [1, 5–7] Parents are frequently concerned by the appearance of children’s feet and worried that their child’s future will be impacted by deformity and pain [1, 5–7]. It has been established that adults with flexible flat feet have a significantly increased likelihood of reporting back or lower limb pain [8, 9], foot pain [10, 11] hallux abducto-valgus [12], callus, hammertoes and degenerative joint disease [13].

The question of the paediatric flat foot markedly divides clinical opinion [14]. Whilst the child’s foot is expected to be flat, there is currently no consensus as to *how flat* the foot should be. Furthermore, whilst feet are observed to decrease in flatness with increasing age, it is not known *how flat* they should be at each advancing year. In fact, no consensus could be found on what age foot postures should cease to change any further. A long held clinical opinion is that mature foot posture is reached between 7 and 10 years of age [1]. However, as a result of paucity in consensus, “the experienced clinician’s discretion” [15] currently guides the decision on whether intervention into paediatric flat foot is required. It is therefore understandable that the decision “to treat or not to treat” [15] remains controversial.

This controversy may be due, in part, to concerns in identifying when a flatfoot is ‘outside of typical’ development. Indeed, to correctly identify abnormal foot posture and therefore manage appropriately, characteristics of “typical” foot posture must be clearly defined. Currently, there is no gold standard assessment method for measuring foot posture in a clinical setting, with a broad array of measurements used. Given the lack of consensus on what constitutes typical development of the paediatric foot; the objective of this systematic review is to define the postural characteristics of the paediatric foot across the ages, and define the measures used to report the foot posture data. This systematic review, to the best of our knowledge, will be the first complete compilation of children’s foot posture data to date.

## Methods

### Review construction

To ensure a standardised approach to the construction of this review, the PRISMA protocol was used [16]. The PRISMA statement is an evidence-based minimum set of items for reporting in systematic reviews and meta-analyses [16]. This review takes the form of a descriptive, comparative analysis, as the studies present epidemiological data, of a cross sectional design.

### Search strategy

Electronic databases Medline, Embase, AMED, CINAHL, Cochrane, Scopus and Web of Science were searched from inception to December 2016. The search strategy expanded and combined key terms pertaining to the concepts; ‘foot’ and ‘child’ and ‘measurement’. Within MEDLINE, Embase and AMED; the following medical subject headings were applied to child*; diagnosis, epidemiology, genetics, growth and development, pathogenicity, rehabilitation, surgery, therapy. The search strategy for Medline, Embase and AMED is presented in Additional file 1. One author further searched the reference lists of identified studies to identify any additional studies.

### Study selection

Studies needed to be of a quantitative design (inclusive of but not limited to; randomised controlled trials, case-cohorts and observational studies) pertaining to the postural development of the child and adolescent foot, available in full text and published within a peer reviewed journal. No date or language restrictions were applied. The population needed to be a healthy, non-pathological (excluding all neurological, rheumatic and connective tissues disorders), asymptomatic (nil lower limb pain) human population, aged approximately 12 months (included at age of independent gait development) to 18 years of age, with no known history of lower limb surgery. Outcome measures of interest were inclusive of all static, weight-bearing, structural and/or postural measures of the foot; excluding dynamic gait data, joint range of motions, plantar pressures and simple morphological data (length, width). All initial ‘search hits’ were screened for relevance by assessing both the title and abstract by one reviewer (HU). Studies deemed ‘potentially relevant’ were then further screened by reviewing full texts by two reviewers independently (HU and RC). Results of the independent reviews were then collated and where inclusion was agreed upon by both authors the study was included. Any discrepancies in opinion were discussed until consensus was reached.

### Data collection and analysis

Data extraction was completed by one author (HU) into a purpose built data extraction file (Microsoft Excel 2010). Data items were inclusive of; study characteristics, bibliographic data, number and demographics of participants, outcome measures, foot posture results and data required for the risk of bias appraisal. Due to included studies being of an epidemiological, observational design (cross-sectional or case series), data were appropriate for inclusion if the outcome measure had been reported for the whole study group (population) and if mean and standard deviation (SD) data were reported. Data were not deemed appropriate if the study had only reported the outcome measure for specific groupings (for example normal weight vs obese groups) or if data were only reported graphically. Additional data were requested electronically (email correspondence) from authors where required.

### Risk of bias and quality appraisal

The Australian National Health and Medical Research Centre designation of levels of evidence – *Aetiology research question;* was used to allocate the methodological design of the included studies [17]. The Epidemiological Appraisal Instrument (EAI) was used to assess the risk of bias of the included studies [18]. The EAI has been validated for the appraisal of observational studies [18]. The EAI contains 43 questions for which the response combinations include ‘Yes’, ‘No’, ‘Partial’, ‘Not Applicable’ and ‘Unable to Determine’. A total of 13 domains not applicable to cross sectional studies were removed, leaving 30 domains to assess risk of bias. Nix et al. used the EAI within their systematic review of cross sectional studies in which the following quantitative scoring system was applied; “Yes” (score = 2), “Partial” (score = 1), “No” (score = 0), “Unable to determine” (score = 0) and ‘Not Applicable’ (removed from scoring) [19]. An average score is then calculated for each study, ranging between 0 and 2 [19]. For the purpose of publication the same scoring system proposed by Nix et al. was applied here within [19]. The EAI was carried out but two authors independently (HU and RS). Where consensus was not reached, a third reviewer was applied (RC).

### Statistical methods

To allow descriptive comparison of foot posture measures, 95% confidence intervals were calculated from the mean and standard deviation (SD) data reported within the studies. Data were calculated and graphically displayed using Microsoft Excel (2010). Observational interpretation of the graphs was then reported. Where standard deviation data were not supplied, the standard method of approximating this data was used (SD ≈ (Max-Min)/4) [20]. Where data collection was described to include both left and right feet of a single study participant, the total sample size (n) was adjusted to reflect true ‘feet’ count, rather than number of participants. This ensures the independence assumption of statistical analysis proposed by Menz [21].

## Results

### Study selection

**Fig. 1:**
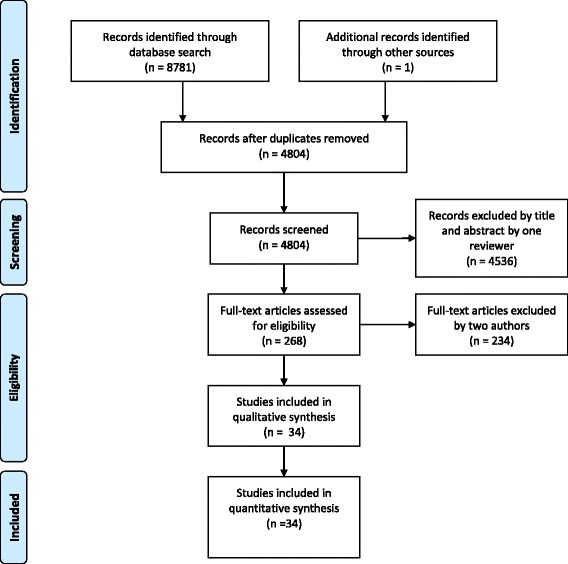
Study selection flow diagram

A total of 8781 papers were initially identified (Fig. 1). Once duplicates were removed, one reviewer (HU) screened 4804 papers for relevance by title and abstract. Two reviewers (HU and RC) then independently assessed the full-text of the remaining 268 studies. Consensus was reached for a total inclusion of 34 studies. Consensus was reached without the need of a third independent reviewer. Language translations were required from Spanish, Polish and Italian to English during the selection process. Thirteen authors were contacted via email requesting additional data that would have enabled their study to be included within the review. Five authors supplied additional data, one author declined to provide additional data, one author provided an alternative study with the same population data presented in a different format, and no response was obtained for the six remaining author requests.

### Study characteristics

The characteristics of all 34 studies are presented within Table 1. Studies were of an epidemiological, cross sectional design presenting foot posture data of children aged from 10 months to 18 years of age. Five studies present longitudinal data ranging from a 12 month assessment period to a 9 year follow-up. Data publication ranged from as early as 1987 to 2016. Nineteen countries are represented throughout the studies providing data for populations of Middle Eastern, North and South American, Asian, African, European and Australian children (Table 1). Significantly large population data sets are included by both Muller et al. [4] and Waesda et al. [22], with 7788 and 10,155 participants respectively. The German population presented within the studies by Bertsch et al., Bosch et al., Sacco et al. and Unger et al. are the same population that were followed longitudinally over a 9 year period [23–26]. The repetition of this data set needs to be considered when reviewing these four studies.

**Table 1:** Study characteristics

Authors	Study Type	Study aim	Population	N Participants (Boys:Girls)	Age range in years (mean ± SD)	Foot posture measures
Bertsch et al. [23]	Longitudinal Cohort	Evaluate plantar pressure data in infants to understand the maturation of the lower extremity and therefore differentiate pathological disorders	German infants, new walkers (*Data collected longitudinally over 12 months*)	42 (20:22)	10–17 *months* (14.8 ± 1.8) *months* at first exam	Foot shape index % (midfoot width/length)
Bosch et al. [24]	Longitudinal Cohort	Establish a plantar pressure database of infants for analysis of individual clinical cases	German infants, new walkers (*Data collected longitudinally over 4 years*)	43–90 (NR)	NR (15.3 ± 2.3)*months* at first exam	Foot form index % (midfoot width/length)
Chang et al. [36]	Cross-sectional	To use a 3D scanner to evaluate the arch of pre-school children and describe the flexibility of the arch	Taiwanese kindergarten children	44 (24:20)	2–6 (NR)	Navicular height computed from ‘Peripher 3D Scanner’
Delgado-Abellán et al. [38]	Cross-sectional	To analyse age and gender differences in foot morphology in Spanish school aged children	Spanish school children	1031 (497:534)	6–12 (NR)	Arch height computed from 3D foot digitiser
Didia et al. [27]	Cross-sectional	To create a data base of foot arch characteristics of a Nigerian population	Nigerian school children	990 (458:532)	5–14 (8.6 ± 1.9)^a^	Contact index II
Dowling et al. [62]	Cross-sectional	To determine whether a pedograph could be used to predict plantar pressures of the feet of primary school children	Australian, pre-pubertal children	51 (22:29)	NR (8.4 ± 1.0)	Clarke’s angle Chippaux-Smirak index
El et al. [45]	Cross-sectional	To analyse the longitudinal arch morphology and related factors including hypermobility, age, gender and rearfoot alignment in primary school children	Turkish primary school children	579 (299:280)	6–12 (9.23 ± 1.66)	Rearfoot angle Staheli arch index
Evans [40]	Cross-sectional	To investigate the relationship between flat foot posture and body weight and related anthropometric measurements in school aged children	Australian, primary school children	140 (68:72)	7–10 (8.71 ± 0.91)	FPI-6
Evans and Karimi [42]	Cross-sectional	Examine the relationship between body mass index and foot posture in children	Five data sets including Australian and United Kingdom children	728 (375:353)	3–15 (9.07 ± 2.38)	FPI-6
Forriol and Pascual [28]	Cross-sectional	To investigate the development of the footprint according to age, gender, growth and foot type	Spanish children	1676 (663:1013)	3–17 (NR)	Clarke’s angle Chippaux-Smirak index
Gijon-Nogueron et al. [43]	Cross-sectional	To establish normative FPI-6 reference values for children aged 6–11 years	Spanish children	1762 (863:899)	6–11 (8.28 ± 1.72)	FPI-6
Gill et al. [48]	Cross-sectional	Examine the relationship between foot arch height and walking characteristics in children and adults	American children	254 (121:133) 18 (10:8)	2–17 (9.13 ± 3.26) 4–8 (6.22 ± 1.26)	Chippaux-Smirak index Keimig index
Gilmour and Burns [29]	Cross-sectional	Examine the influence of gender, limb preference and body weight in relation to the medial longitudinal arch in children	Australian children	272 (128:144)	5.5–10.9 (8.4 ± 1.7)	Arch index Navicular height
Hallemans et al. [46]	Cross-sectional	To perform a longitudinal study investigating foot function changes within the first 5 months of walking	Belgian toddlers	10 (3:7)	10–15 (12.6 ± 1.7) *months*	Foot form index % (width/length)
Hawke et al. [41]	Cross-sectional	A post hoc analysis to explore the relationships between foot posture, flexibility and body mass in children	New Zealander children; 90% Caucasian, 7% Asian, 3% Maori.	30 (10:20)	7–15 (10.7 ± 2.3)	FPI-6
Igbigbi and Msamati [32]	Cross-sectional	To determine the arch index, classify the arch type and report the incidence of pes planus amongst the Malawian population	Indigenous Malawian teenagers	305 (139:166)	13–17 (NR)	Arch index
Igbigbi et al. [33]	Cross-sectional	To determine the AI, classify the arch type and report the incidence of pes planus amongst a Kenyan and Tanzanian population	Kenyan teenagers Tanzanian teenagers	314 (174:140) 249 (135:114)	13–17 (NR)	Arch index
Jankowicz-Szymanska and Mikolajczyk [49]	Longitudinal Cohort	To investigate the changes in the height of the medial longitudinal and transverse arches of the foot over a 2-year follow-up	Polish kindergarten children	207 (102:105)	3.5–4.49 (NR) at first exam	Clarke’s angle Gamma angle
Mauch et al. [61]	Cross-sectional	To investigate the shape of children’s feet and assess if a difference exists between the feet of German and Australian children	Australian pre and primary school children German pre and primary school children	86 (34:52) 419 (190:229) 86 (34:52) 419 (190:229)	3–5 (4.3 ± 0.6) 7–12 (9.6 ± 1.4) 3–5 (4.2 ± 0.7) 7–12 (9.6 ± 1.4)	Clarke’s angle Chippaux-Smirak index
Jankowicz-Szymanska and Mikolajczyk [49]	Cross-sectional	To assess the somatic features and to determine the correlation between skin fold thickness and MLA height and knee position children	Polish primary school children	90 (45:45)	7 (NR)	Clarke’s angle
Morita et al. [63]		To investigate muscle strength and arch height and explore the relationships between these measures and lower limb physical performance	Japanese primary school children	301 (146:155)	Third grade *n = 158* (8.6 ± 0.5) Fifth grade *N = 143* (10.6 ± 0.5)	Foot arch height (FAH) – *height of navicular tuberosity to ground* Foot arch index – (*FAH/foot length* × *100)*
Morrison et al. [37]	Cross-sectional	To evaluate the impact of excessive body mass on the anthropometric structure of the prepubescent foot	Scottish primary school children	200 (90:110)	9–12 (10.4 ± 0.9) ♂ (10.1 ± 0.8) ♀	Navicular height
Muller et al. [4]	Cross-sectional	To measure the static and dynamic foot characteristics in infants and children to establish foot structure and function in different age groups	German children	7788 (3738:4050)	1–13 (7.2 ± 2.9)	Arch index
Nikolaidou and Boudolos [44]	Cross-sectional	To establish a footprint-based classification technique for the rational classification of foot types in young schoolchildren	Greek school children	132 (67:65)	NR (10.4 ± 0.9)	Arch index Martirosov’s K index Chippaux-Smirak index Clarke’s angle
Pfeiffer et al. [3]	Cross-sectional	To establish the prevalence of flat foot in a population of 3–6 year olds, evaluating cofactors including age, gender and weight	Austrian children	835 (424:411)	3–6 (4.4 ± 0.9)	Rearfoot angle
Pinto et al. [47]	Cross-sectional	To evaluate whether a footprint taken during the Jack test could be quantified in children 2–5 years	Brazilian children	60 (35:25)	2–5 (3.4 ± NR)	Volpon footprint Valenti footprint
Redmond et al. [39]	Cross-sectional	To establish normative FPI reference values for use in research and clinical decision making	3 accumulated data sets; data sets inclusive of 4, 5 and 9 Ethnicity un-known	397	3–17 (8.5 ± NR)	FPI-6
Sacco et al. [25]	Longitudinal Cohort	To compare the anthropometric characteristics of children’s feet from 3–10 years between German and Brazilian populations *(Data reordered longitudinally for 9 years)*	German children Brazilian children	51–94 (NR) 391 (183:208)	3–10 (NR) 3–10 (NR)	Chippaux-Smirak index Staheli arch index
Sadeghi-Demneh et al. [35]	Cross-sectional	Determine the prevalence of flatfoot among elementary and secondary school children. Evaluating also age, gender, joint laxity and obesity	Iranian children	667 (340:327)	7–14 (10.6 ± 2.3)	Arch index Rearfoot angle Arch angle
Tong and Kong [30]	Longitudinal cohort	To examine the medial longitudinal arch of children during development and explore the relationship between different footwear use	Singaporean children	111 (52:59)	(6.9 ± 0.3)	Arch index
Sobel et al. [34]	Cross-sectional	Determine the rearfoot angle in children in different age groups	African American children	150 (52:98)	6–12 (10.79 ± 2.75)	Rearfoot angle
Tudor et al. [64]	Cross-sectional	To determine if there is an association between the severity of foot flatness and motor skills necessary for sport performance	Croatian children	218 (106:112)	11–15 (13.07 ± 1.24)	Arch index
Unger and Rosenbaum [26]	Cross-sectional	To evaluate the foot shape statically and dynamically during walking	German Infant new walkers	42 (20:22)	NR	Arch index Foot shape index % (width/length)
Waseda et al. [22]	Cross-sectional	To establish standardised values of foot length and arch height in children and adolescents	Japanese school children	10,155 (5311:4844)	6–18 (NR)	Navicular height Arch height ratio

### Risk of bias

Application of the EAI across all 35 studies can be seen within Additional file 2. The domain numbers have remained consistent with the original EAI document despite the removal of 13 non-applicable domains [18]. Domains that consistently reported the lowest compliance were an inadequate description of the sampling frame, participation rates, inadequate provision of statistical parameters, not randomising groups, not concealing randomisation, not blinding the observers, not blinding participants to their grouping and the inability to determine generalisability to the greater population (3%, 3%, 3%, 0%, 0%, 0%, 0% and 0% respectively), (Additional file 2). Conversely, compliance was good for domains 1–4 with the adequate description of the study aims, exposure variables, main outcomes and the study design (97%, 97%, 97% and 100% respectively). Where grouping was applicable, there was 100% compliance with domains 27 and 33 suggesting that both exposure methods and outcome variables were standardised. When converting each paper’s quality appraisal performance to an overall score between 0 and 2, less than half of the papers scored ≥1 (43%). The highest quality score was attained by Didia et al., 90%, with the lowest recorded by Forriol et al., 30% [27, 28].

### Outcome measures

Sixteen different outcome measures were used to measure foot posture. Footprint analysis was the most commonly used assessment method with a total of 10 different footprint-based measures utilised. These include the arch index (AI), Staheli arch index (SAI), Footform index, Clarke’s angle (or alpha angle), Chippaux-Smirak index (CSI), Contact index II (or Volpon index), Martirosov’s K index, Valenti index, Gamma angle and the Keimig index. The remaining six measures used were navicular height, arch height ratio, arch angle, the Foot Posture Index 6 (FPI-6), rearfoot angle (also reported as the hindfoot angle or resting calcaneal stance position) and 3D arch angle. Additional file 3 contains the measurement procedures and descriptions for all comparative foot posture measures found within this review.

### Foot posture comparisons

The epidemiological, cross sectional data reported allows for graphical, comparative analysis only. Comparisons in data sets were available for the AI, CSI, SAI, navicular height, arch height ratio, the FPI-6, Clarke’s angle, Footform Index, Contact Index II and rearfoot angle. Foot posture categories relevant to each measure are noted within each graph where appropriate with measurement descriptions within Additional file 3. All descriptive terms used to categorise and describe foot postures herein below are therefore reported exactly as the measure has termed their use.

#### The arch index

**Fig. 2:**
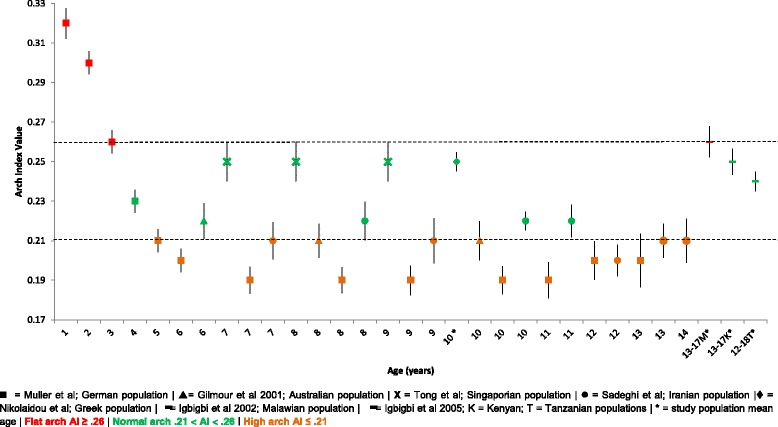
Arch index Vs Age: Mean, 95% CI Comparison Graph

Mean, SD and 95% CI, comparative data for the AI from 1 to 18 years of age for German, Australian, Singaporean, Greek, Malawian, Kenyan and Tanzanian populations is presented within Fig. 2. Data from 1 to 3 years of age reports feet that are consistent with ‘flat arched’ feet, albeit from only one German population source [4]. From 4 to 11 years the data reports both a degree of normal and high arched feet from a combined population of German, Australian, Singaporean and Iranian children [4, 29–31]. Igbigbi’s African populations of children aged 12–18 years of age predominately demonstrate normal arched feet with the Malawian population demonstrating a higher AI value [32, 33].

#### Rearfoot angle

**Fig. 3:**
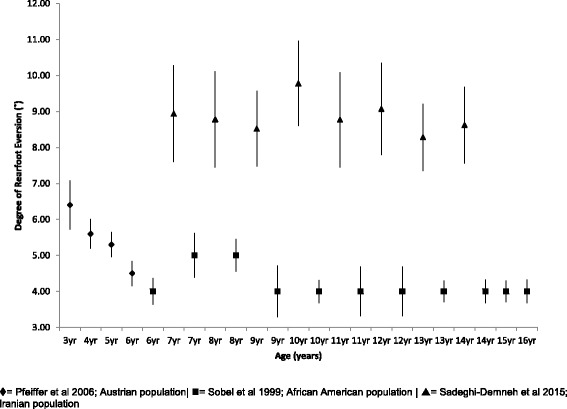
Rearfoot angle (°) Vs Age: Mean, 95% CI Comparison Graph

Mean, SD and 95% CI, comparative rearfoot angle data between Pfeiffer et al., Sobel et al. and Sadeghi-Demneh et al. for children aged 3–16 years of age can be seen within Fig. 3 [3, 34, 35]. Consistently higher rearfoot valgus angles can be seen for the Iranian population of children aged 7–14 years in comparison to the African American population of children spanning the same age years. After an initial decline in rearfoot angle from 3 to 6 years of age within the Austrian population, the rearfoot angle then remains stable from 6 to 16 years of age.

#### The Chippaux-Smirak index

**Fig. 4:**
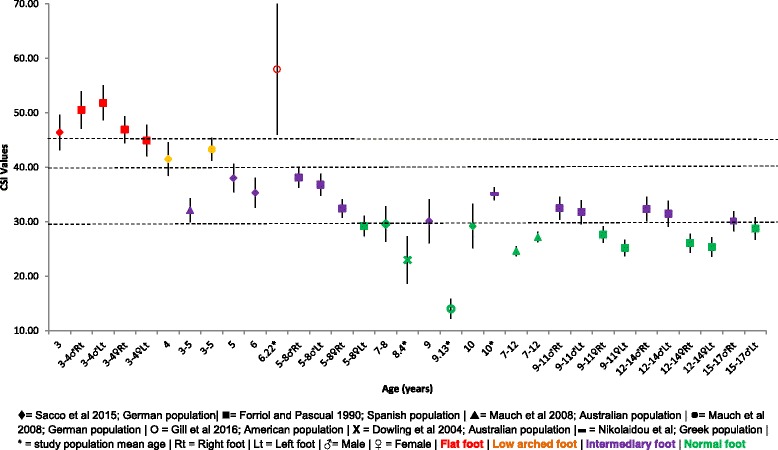
Chippaux-Smirak index (%) Vs Age: Mean, 95% CI Comparison Graph

Comparative mean, SD and 95% CI CSI data for children aged 3–17 years of age is presented in Fig. 4. Overall data progression shows an increase in medial arch height steadily from 3 to 8 years of age. Both Sacco et al. and Forriol and Pascual et al. note a ‘flat foot’ type for children aged 3–4 years [25, 28]. Although not consistent, the data suggests a ‘normal foot’ posture being reached around 8 years of age, with Forriol and Pascual’s Spanish population denoting a consistently ‘lower’ foot arch in males when compared to females of the same age [28].

#### Navicular height

**Fig. 5:**
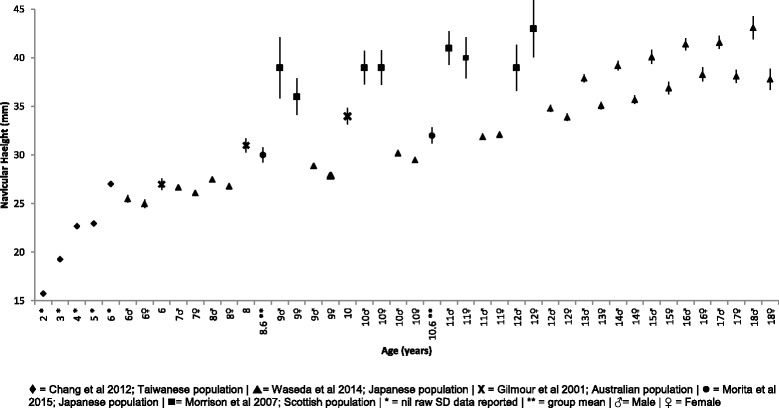
Navicular height (mm) Vs Age: Mean, 95% CI Comparison Graph

Mean, SD and 95% CI comparative navicular height data presented in Fig. 5 increases throughout growth from 2 to 18 years. Of note, Chang et al. reported mean data but did not report standard deviations, thus, confidence intervals were not able to be determined [36]. With the exception of Morrison et al. at 12 years and Waseda et al. at 11 years, all data points demonstrate male navicular height to be higher than female navicular height [22, 37].

#### Arch height ratio

**Fig. 6:**
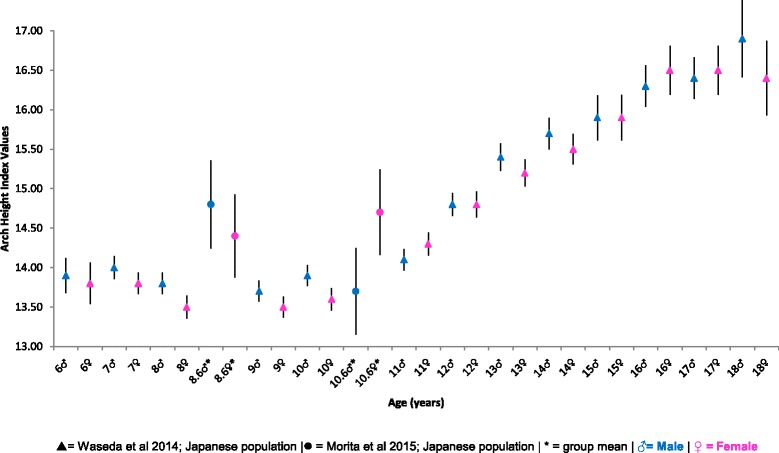
Arch height ratio Vs Age: Mean, 95% CI Comparison Graph

Navicular height is known to be associated with foot length, thus normalising the navicular height by foot length provides an arch height ratio; (navicular height/ft length × 100) [38]. Figure 6 reports the comparative mean, SD and 95% CI arch height ratio for Japanese populations of children aged 6–18 years of age. Generally the graph shows a more stable increase in this ratio between the ages of 6–10 years; followed by a more rapid increase from 11 to 18 years of age. A trend of the males’ arch height ratios being predominately higher than females can also be noted.

#### Foot posture index-6

**Fig. 7:**
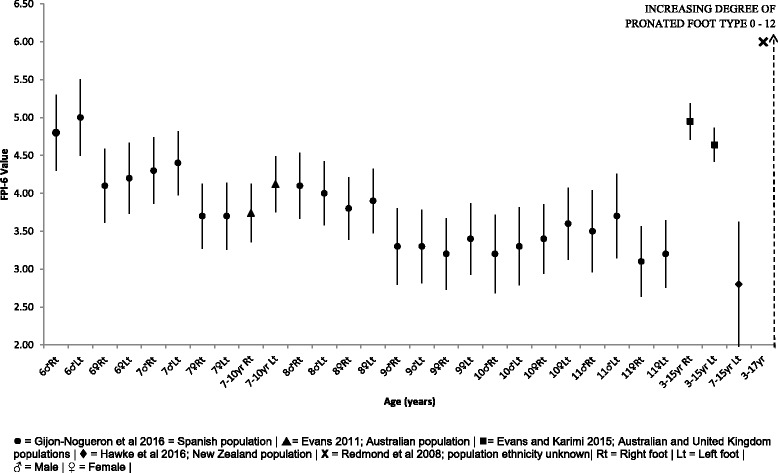
FPI-6 Vs Age: Mean, 95% CI Comparison Graph

Comparative mean, SD and 95% CI FPI-6 data is presented within Fig. 7. The FPI-6 represents the only composite foot measure within this review, capturing multiple segments of the foot within the one overall score [39]. Overall the FPI-6 data confirms that the foot posture of healthy and typically developing children aged 3–17 years of age is that of a pronated foot type [30, 39–42]. Only one Spanish population presents FPI-6 data at discrete yearly increments from age 6 to 11 years, in which very little change in foot posture can be observed over this 5 year period [43].

#### Clarke’s angle

**Fig. 8:**
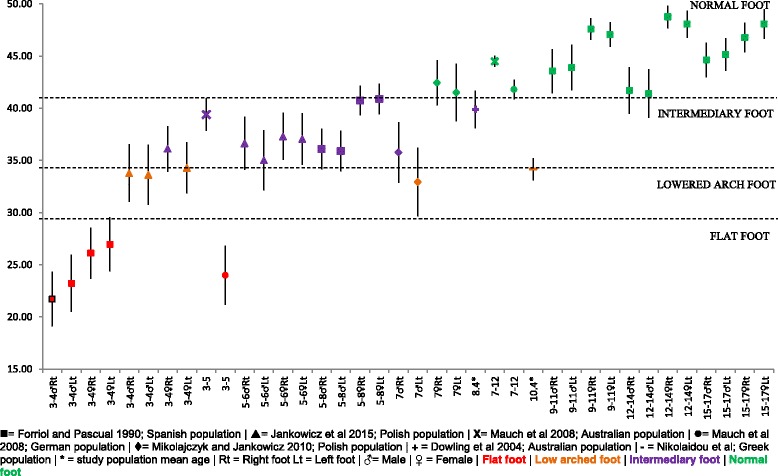
Clarke’s angle (°) Vs Age: Mean, 95% CI Comparison Graph

Figure 8 presents comparative mean, SD and 95% CI data of the Clarke’s angle for children aged 3–17 years of age of Spanish, Polish, Australian, German and Greek descent. Four categories of foot types are categorised from the Clarke’s angle; with the graph showing a progression from the ‘flat foot’ category to the ‘normal’ foot type by around 7 years of age [44]. This progression holds true despite the ethnicity of the population reported, with no significant outliers noted.

#### The Staheli arch index

**Fig. 9:**
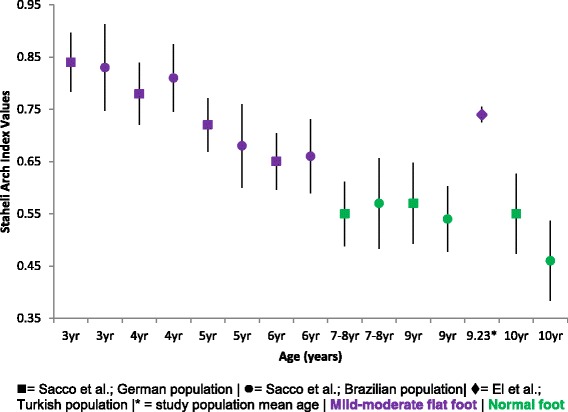
Staheli arch index Vs Age: Mean, 95% CI Comparison Graph

Figure 9 presents the comparative mean, SD and 95% CI SAI results across Sacco and colleagues population of both German and Brazilian children aged 3–10 years of age [25]. This data depicts a ‘mild-moderate flat foot’ at age 3–6 years progressing to a ‘normal foot’ from 7 to 10 years of age. El et al. demonstrates a ‘mild-moderate flat foot’ type for Turkish children of a mean age of 9.23 years [45]. Overall the trend of data shows a consistent decrease in SAI values, thus an increasing medial longitudinal arch height from 3 to 10 years, potentially stabilising from 7 years of age.

#### Footform index

**Fig. 10:**
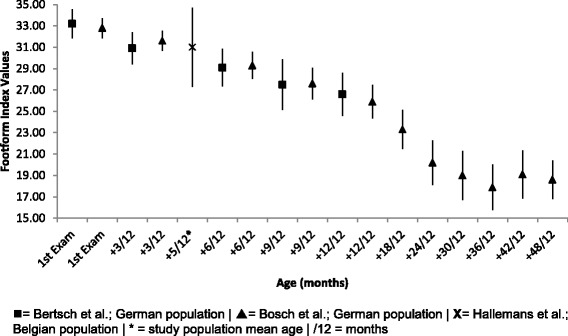
Footform index Vs Age: Mean, 95% CI Comparison Graph

Comparative mean, SD and 95% CI Footform index data for children from initiation of independent walking to 48 months (4 years) post initiation is presented in Fig. 10. The data presented shows within the German and Belgian populations a stable decrease in this measure over 4 years, depicting an increase in arch height [23, 24, 46].

#### Contact index II / Volpon index

**Fig. 11:**
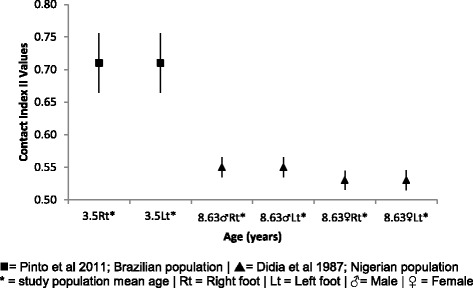
Contact index II Vs Age: Mean, 95% CI Comparison Graph

Little can be inferred from comparative mean, SD and 95% CI data presented within Fig. 11, with only two authors reporting data from the Contact Index II [27, 47]. Data is suggestive only of a higher index value at 3.5 years of age and a lower index value at 8.63 years of age.

#### Foot measures with nil comparison

No comparisons were possible for Martirosov’s k index, Valenti index, Keimig indices, 3D arch height, arch angle and gamma angle. Arch height was measured with a 3D foot digitiser by Delgado-Abellan and colleagues, described as the distance from the ground plane to the most prominent point of the arch [38]. This reported arch height in children aged 6–12 years increased, with no difference in the arch heights of boys and girls once arch height was normalised to foot length. The Keimig index (KI) reported by Gill et al. seeks to quantify the departure of the plantar surface of the foot from the ground surface. Thus, a higher KI value represents a higher arch, whilst a lower KI value represents a lower arch [48]. Using this novel measure Gill and colleagues reported a mean KI value of 0.59 (±0.26) for children aged 2–17 years of age and 0.58 (±0.26) for children aged 4–8 years of age.

Nikolaidou and Boudolos assessed the feet of Greek children with a mean age of 10.4 years (±0.9) using the Martirosov’s k index [44]. The following proportions for foot type were determined in their cohort; 0% high arch, 23% normal, 46% low arch and 31% flat foot type. Pinto et al. present the data for the Valenti index, another foot print based indices [47]. In their Brazilian cohort of children aged 2–5 years of age the Valenti index was reported to be a mean of 0.54 (0.17) for left feet and 0.55 (0.17) for right feet. The gamma angle reported by Jankowicz-Szymanska and Mikolajczyk was described as the angle made between the tangent and the medial and lateral edges of the foot [49]. The change in this angle over the two-year follow up represented an increase in the medial longitudinal arch for boys and a decrease in the height of the longitudinal arch for girls aged approximately 3.5 to 6.5 years of age [49].

The arch angle described by Sadeghi-Demneh et al. produces an angle from the bisection of lines connecting the navicular to the medial malleolus; and the navicular to the head of the first metatarsal. This angle was shown to average 131° ± 6° in Iranian children 7–14 years of age. The rate of flat feet reduced with increasing age, with the highest proportion at 7 years of age and lowest incidence at 13 years of age.

## Discussion

In order for clinicians to recognise and correctly manage ‘abnormally’ developing paediatric feet, first there must be consensus as to what constitutes ‘typically’ developing paediatric feet. This review sought to describe this foot posture according to the current literature base. As a result this review has provided a summary of foot posture values of a healthy, typically developing population of children and adolescents.

The prevalence estimates of flat feet in children have been suggested to range from 0.6–77.9%, with consistent trends of reducing prevalence with increasing age [3]. This broad variation in prevelence estimates could be explained with the lack of consensus in the definition of flat feet and the lack of consistency in the measurement of foot posture, as has been demonstrated wihtin this review. It is clinically accepted that all typically, developing children are born with flexible flat feet, progressively developing a medial longitudinal arch during the first decade of life [1, 2]. The data presented in this review shows that healthy, typically developing children can be expected to have a flat foot type during their development. Specifically reflected within this review at 1–3 years, 3–8 years, 3–7 years and 3–6 years by the AI, CSI, Clarke’s angle and SAI. The same four measures report the ascension to ‘normality’ reached by 4 years, 5–8 years, 7 years and 7–8 years respectively. Whilst these authors use the term ‘normal’ to represent the foot posture at these ages above, it would appear to be an improper description of the child’s foot posture, given that these same healthy, typically developing children were also observed to have a ‘flat foot’ posture at the years prior to this. Simply stated ‘normal foot posture’ can, and does, equate to ‘flat’ as foot posture is age dependent. These foot postures aren’t actually ascending to ‘normality’ they are ‘normally flat’ by a varying amount, with ‘flatness’ reducing with increasing age, not simply flat or normal as these foot measures rudimentarily categorise.

Foot measures that provide a categorical outcome, use varying descriptive terms for the foot posture assessed, inclusive of; flat arch, low arched foot, mild-moderate flat foot, intermediary foot, normal foot and high arch. These authors suggest, that the discussion of ‘normality’ in regards to the paediatric foot posture could align more readily with reference percentile values, in keeping with the majority of other developmental children’s assessment methods. The assessment of children’s weight, height, head circumference and motor milestones are readily reported against percentile values. Whereby, not only the percentile score at any one point in time is of importance, but indeed the consistent trajectory of that measure over time offers a clear, validated measure of change. Future foot posture research in paediatrics should also provide data in-line with these other commonly reported children’s measures,

The question of what age should one consider the child’s foot to cease arch development, or rather, cease to be flat, may be a more important conclusion to consider. Three methods reported in the literature by which the child’s foot could be considered *mature* include; cessation of growth, closure of growth plates and stabilisation of posture [50–54]. The length of the foot is suggested by Leung et al. to increase linearly in girls from 4 to 13 years of age and in boys from 4 to 14 years of age [50]. Similarly, Liu and colleagues suggest that the cessation of foot length occurs at 15.58 (±1.26) years for boys and 13.56 (±1.17) years for girls [51]. Growth plate fusion in children’s feet is expected to be complete by 16 years of age [52]. Anecdotally, podiatrists consider a child’s foot posture to become static at around 7–8 years of age, whilst this is generally accepted, no original reference for this information can be found [53]. Onodera and colleagues in a study comparing the agreement between multiple footprint indices concluded that the maturation of the medial longitudinal arch continues after 6 years of age, at a slower velocity until 10 years of age [54]. At which time the majority of the children’s footprints had reached ‘normal’, with minimal variation [54].

Whilst a uniform approach to the description of children’s foot posture is required, so too is a uniform foot assessment method [5]. Sixteen different measures were presented within this systematic review, with varying degrees of comparison available. The most readily reported outcome measures were footprint-based techniques. The use of a footprint based measure to represent a postural foot position has caused significant conjecture in the literature and indeed initiated the ‘Flat or Fat?’ debate [55]. Simply categorising the arch around dichotomous pathologies of either flat, normal, or high treats the arch as a simple uni-planar structure and in doing so disregards the complexity and multi-planar motion of the foot [48]. Gill and colleagues aptly demonstrated that feet with the same CSI and KI values could have vastly different foot prints and functional gait profiles [48]. Thus foot print-based measures may not be specific enough to capture the significant postural differences that feet can present with.

Of the remaining measures, arguably the most clinically popular include; navicular height, rearfoot angle and the FPI-6. Navicular height which has been shown to be a reliable measure in an adult population, demonstrates poor reliability within a paediatric population, particularly so in the very young and only useful when the measure is normalised to foot length [56, 57]. Anecdotally, rearfoot angle is used and taught widely amongst podiatrists, however, the functionality of its use may be limited, being that it is a single plane measurement used to infer the position of the subtalar joint which is a tri-planar joint and is prone to substantial measurement error [58]. Once again, as per the previous foot print measures, these measures on their own may not be specific enough to take into account the entire complexity of a flat foot type.

The FPI-6 is a multi-planar measurement process, which has also demonstrated good reliability and ease of use [39, 59]. Whilst a useful clinical measure, the FPI-6 results presented within this systematic review are less useful owing to the large age spread of children included within each data point (7–10, 3–15 and 3–17 year olds). Any inferences from this data should be made with caution as the FPI-6 score would be predicted to change with each year of childhood, as has been shown with the other foot posture measures.

The results of this systematic review do suggest a disproportionate usage of footprint-based measures when compared to clinical foot posture measures such as the FPI-6. Footprint measures were extensively reported within the body of foot posture literature, yet, in these authors experiences at least, are not frequently used within clinical practice. Conversely, the FPI-6, is frequently used within clinical practice, evidenced by its inclusion in the Gait and Lower Limb Observation of Paediatrics (GALLOP) tool, which was based on expert consensus amongst paediatric physiotherapists and podiatrists [60]. This may suggest a disparity between how paediatric flat foot is measured in the literature and how it is assessed in clinical practice. Furthermore, the results of this systematic review suggest that very little consensus exists within the literature on which foot posture measures best assess the paediatric foot. With the research currently available no recommendations for clinical practice can be inferred.

Importantly, a foot posture measure by itself may not fully represent foot function and requires placement within the clinical context. Indeed, the structurally *abnormal* foot can present asymptomatically, whilst a seemingly structurally *normal* foot can present symptomatically. Working within paediatrics requires the clinician to assess the whole child rather than simply the aesthetics of their foot posture. With this in mind, the data within this systematic review reports static, weight-bearing foot structure of the paediatric foot only, and cannot infer context of dynamic function or management of the paediatric foot. The purpose of this systematic review was not to direct when intervention is necessitated. The paediatric flat foot proforma provides the first, and to the best of our knowledge, and only evidence based clinical care pathway towards the management of the paediatric foot for children aged 7 years and younger [15]. This proforma is the first of its kind to marry subjective patient information with clinical observations and directs the clinician towards an appropriate management pathway.

This systematic review itself is not without its own limitations, namely, the small literature base on which these results have been drawn from, small sample sizes within the included studies and the accuracy of the measures used have not been considered. More specifically, the validity of the foot posture measurements has not been reported. A systematic review by nature simply collates and reports the findings of the existing literature base. The authors also recognise that the provision of an overall ‘summed score’ for critical appraisal tools was reported to allow for consistency in the use of the EAI as per Nix et al. [19]. The reader needs to be cautious in using these summed scores as it assumes equal weighting of the domains, which would not be an accurate assumption.

Just as foot posture is determined by age, the following known covariates have not been adequately explored within this systematic review to enable any conclusions to be drawn, specifically; sex [3, 24, 26, 45, 50, 51, 61] and BMI [3, 61]. Whilst ethnicity and/or country of origin was reported, no relationship that may exist between foot posture and ethnicity were formally explored.

As a result of this systematic review many questions have been raised. Firstly, which of these foot measures, if any, should be used to assess the posture of the developing foot? Secondly, what level of importance, if any, should be placed on the static posture of the developing foot, in the notable absence of functional and clinical data? And finally, how should *abnormal* foot postures therefore be recognised? Whilst the desire of clinicians is to appropriately manage their patients, this systematic review has demonstrated a paucity in consensus amongst the literature pertaining to the typically developing paediatric foot, which ultimately reduces the clinicians ability to do so.

## Conclusions

Several important conclusions can be drawn from this systematic review, a compilation of healthy, typically developing children’s feet. The first being that the use of the term *normal* in relation to foot posture is misleading in the categorisation of the paediatric foot, as indeed a flat foot posture is a typical finding at specific ages; flat equals normal. The second conclusion of importance being that the foot posture of the developing child is indeed age dependent and has been shown to change over time. Finally, no firm conclusion could be reached as to which age the foot posture of children ceases to develop further, specifically the medial longitudinal arch, as no two foot measures are comparable. Future research needs to consider the development of guidelines which provide direction on how to measure the paediatric foot, using valid and reliable assessment tools to ensure prevalence reports are appropriate and translatable.

## Additional files

Additional file 1: Medline, Embase and AHMED search strategy. (DOCX 11 kb)

Additional file 2: Epidemiological Appraisal Instrument. (XLSX 16 kb)

Additional file 3: Measurement protocols for all comparative foot posture measures. (DOCX 156 kb)

## Abbreviations

AI: Arch index
CI: Confidence intervals
CSI: Chippaux-Smirak index
EAI: The epidemiological appraisal instrument
FPI-6: Foot posture index 6
GALLOP: Gait and lower limb observation of paediatrics tool
SAI: Staheli arch index
SD: Standard deviation
