# Supplementary material for: The typically developing paediatric foot: how flat should it be? A systematic review
Source: J Foot Ankle Res. 2017 Aug 15;10:37. doi: 10.1186/s13047-017-0218-1 (PMC5558233; doi:10.1186/s13047-017-0218-1)
Supplement: Supplementary file 1 — Medline, Embase and AHMED search strategy. (DOCX 11 kb) [file 13047_2017_218_MOESM1_ESM.docx]

Additional File 1 – Search Strategy

| exp Foot/  (foot or feet or rearfoot or forefoot or midfoot or calcaneus or heel or arch or navicular) | AND |
| --- | --- |
| adolescent/ or child/ or preschool/ or infant/  (p$ediatric or adolescen* or child* or teenager* or toddler) | AND |
| (posture* or biomech* or footprint* or morphology*) |  |
