# Supplementary material for: The typically developing paediatric foot: how flat should it be? A systematic review
Source: J Foot Ankle Res. 2017 Aug 15;10:37. doi: 10.1186/s13047-017-0218-1 (PMC5558233; doi:10.1186/s13047-017-0218-1)
Supplement: Supplementary file 3 — Measurement protocols for all comparative foot posture measures. (DOCX 156 kb) [file 13047_2017_218_MOESM3_ESM.docx]

**Additional File 3** – Measurement protocols for all comparative foot posture measures

**The Arch Index**

**
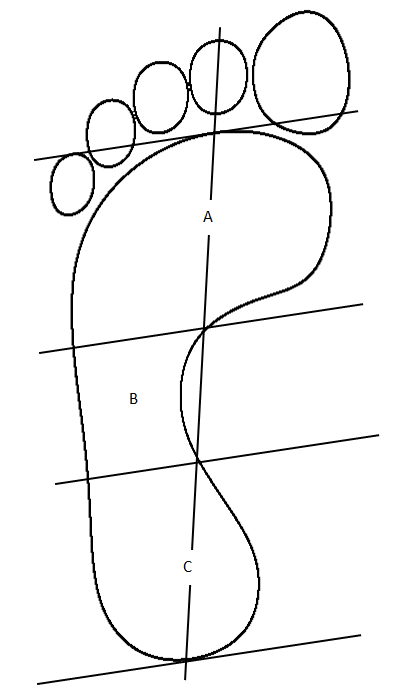
AI** = areaB/(areaA+areaB+areaC)

A high arch index value is indicative of a flat foot type, whilst a small arch index value would be consistent with a high arched foot .

**Chippaux-Smirak Index , Footprint angle (*clarkes angle, alpha angle*) & Staheli index**

**
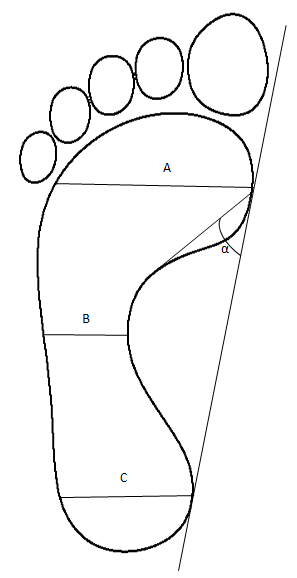
CSI** = widthB/widthA x 100%

Five categories are described using the CSI: 0% = High arch foot; 0.1%>29.9% = normal morphological foot; 30%>39.9% = intermediary foot; 40%>44.9% = lowered arch and 45%≥ = morphological flat foot

**Footprint angle** = α˚

Three categories are described: 0˚>29.9˚ = morphological flatfoot; 30˚>34.9˚ = lower plantar arch and 35˚>41.9˚ = intermediary foot posture

**Staheli arch index** = widthb/widthc x 100%

**Rearfoot Angle**

An angle measured form the intersection of a posterior bisection of the calcaneus and the horizontal (supporting surface). This angle infers the degree of varus (+˚) or valgus (-˚) tilting of the weightbearing rearfoot.

**Navicular Height**

Measured in mm, the height of the navicular tuberosity is measured perpendicularly to the ground.


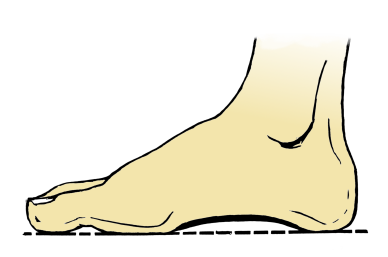


**Arch height ratio**

The navicular height (NH) is divided by the length of the foot (FL). NH/FL x 100

**FPI-6**

Comprised of six criterion based observations of feet in a relaxed, fully weight-bearing position:

1. Palpation of the talus head
2. Observation of the curves above and below the lateral malleolus
3. Extent of inversion/eversion at the calcaneus
4. Talonavicular bulge
5. Congruency of the medial longitudinal arch
6. Extent of abduction/adduction of the forefoot on the rearfoot

Each observation is scored with either a: -2, -1, 0, +1,+2. Scores less than zero are indicative of a supinated alignment and over zero indicivtive of a pronated alignment. Overall scores are added together to give a score from -12 (most supinated) to +12 (most pronated).

**Footform index**

Footform index is calculated from a footprint by measuring the width at the narrowest portion of the midfoot and dividing by the total length of the foot. midfoot width/length x 100%.

**Contact index II/volpon index**

**
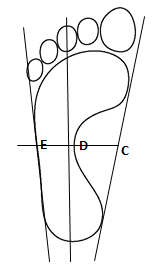
**Measurement of distances DE and CE calculated. Indice is then DE/CE.
